# The use of plant models in deep learning: an application to leaf counting in rosette plants

**DOI:** 10.1186/s13007-018-0273-z

**Published:** 2018-01-18

**Authors:** Jordan Ubbens, Mikolaj Cieslak, Przemyslaw Prusinkiewicz, Ian Stavness

**Affiliations:** 1 University of Saskatchewan, 105 Administration Place, Saskatoon, S7N 5C5 Canada; 2 University of Calgary, 2500 University Dr NW, Calgary, T2N 1N4 Canada

**Keywords:** Phenotyping, Deep learning, Machine learning, 3D plant modeling, L-system

## Abstract

Deep learning presents many opportunities for image-based plant phenotyping. Here we consider the capability of deep convolutional neural networks to perform the leaf counting task. Deep learning techniques typically require large and diverse datasets to learn generalizable models without providing *a priori* an engineered algorithm for performing the task. This requirement is challenging, however, for applications in the plant phenotyping field, where available datasets are often small and the costs associated with generating new data are high. In this work we propose a new method for augmenting plant phenotyping datasets using rendered images of synthetic plants. We demonstrate that the use of high-quality 3D synthetic plants to augment a dataset can improve performance on the leaf counting task. We also show that the ability of the model to generate an arbitrary distribution of phenotypes mitigates the problem of *dataset shift* when training and testing on different datasets. Finally, we show that real and synthetic plants are significantly interchangeable when training a neural network on the leaf counting task.

**Electronic supplementary material:**

The online version of this article (10.1186/s13007-018-0273-z) contains supplementary material, which is available to authorized users.

## Background

Non-destructive, image-based plant phenotyping has emerged as an active area of research in recent years. This is due in part to a gap in capability between genomics and phenomics, as well as the complexity of genotype-to-phenotype mapping [1]. The ability to correlate heritable traits with genetic markers relies on the accurate measurement of phenotypes. In order to achieve statistical power, this measurement typically needs to be done at a large scale which makes measurement by hand intractable. Image-based phenotyping is an important tool for genotype-phenotype association as it allows for the required automation. High-throughout imaging is aided by imaging technologies available in some automated greenhouses [2], as well as low-cost imaging tools which can be made with off-the-shelf parts [3]. An appropriate software environment is also required for the automatic extraction of phenotypic features from the image data. Ideally, such software should be highly automated, scalable, and reliable. Although high-throughput phenotyping is typically conducted in circumstances where the scene can be controlled, for instance on rotating stages in imaging booths, computer vision algorithms should be invariant to changes in the scene if they are to be used in greenhouse or field environments. These algorithms should also take into account other factors, such as the structural variation between different species or accessions, the shape and color of leaves, and the density and geometric eccentricity of the shoots. Therefore, any algorithm that contains parameters which are hand-tuned to a specific collection of plants is at risk of being overly specified.

Unlike engineered computer vision pipelines, deep neural networks learn a representation of the data without image parameters specified by hand. This makes them potentially more robust to different types of variations in the image data, as the network can adapt to be invariant to such differences. However, the transition from hand-engineered computer vision pipelines to deep learning is not without limitations. While so-called “deep” networks have the representational capacity to learn complex models of plant phenotypes, the robustness of these representations relies on the quality and quantity of the training data. In most vision-based tasks where deep learning shows a significant advantage over engineered methods, such as image segmentation, classification, and detection and localization of specific objects in a scene, the size of the dataset is typically on the order of tens of thousands to tens of millions of images [4]. This allows for much variety in the training data, and very robust learned representations as a consequence.

Unfortunately, datasets of plant images, labeled with corresponding phenotypic data, are not yet available on a large scale due to the considerable expense involved in collecting and annotating this type of data. In addition, any supervised machine learning method, including deep learning, requires that the data used to train the model is representative of the data used at test time. Plant phenotyping tasks are vulnerable to such problems with incomplete training data due to the difficulty of generating a dataset in which a comprehensively wide range of phenotypes are represented.

The small size of existing plant phenotyping datasets, the expense of generating new data, and the limitations of naturally-generated datasets motivate the use of an alternative source of data to train deep networks for plant phenotyping tasks. For this purpose we propose the use of synthetic plants—images of computer-generated plant models—to augment datasets of plant images or to be used alone as a large and rich source of training data. Compared to generating new data using real plants, once a model is developed, the generation of new data is essentially without cost. Moreover, models can be parameterized to generate an arbitrary distribution of phenotypes, and ground-truth phenotype labels can be automatically generated without any measurement errors and without any human effort or intervention.

### Deep learning

Deep learning refers to a broad category of machine learning techniques, which typically involve the learning of features in a hierarchical fashion. Such techniques have been shown to be successful in many types of computer vision tasks, including image classification, multi-instance detection, and segmentation [5]. Deep learning is an area of active research, and applications to plant science are still in the early stages. Previous work has shown the advantage of deep learning in complex image-based plant phenotyping tasks over traditional hand-engineered computer vision pipelines for the same task. Such tasks include leaf counting, age estimation, mutant classification [6], plant disease detection and diagnosis from leaf images [7], the classification of fruits and other organs [8], as well as pixel-wise localization of root and shoot tips, and ears [9]. The small body of existing research on deep learning applications in image-based plant phenotyping shows promise for future work in this field.

We trained Convolutional Neural Networks (CNNs) using the open-source Deep Plant Phenomics platform [6] to perform each of the experiments presented in this work. CNNs are often used for classification and regression, where the input data contains some sort of local connectedness, for example, spatially local features in images. A CNN contains one or more convolutional layers, each receiving an input volume and outputting an output volume. An image is considered to be a $n \times m \times 3$ volume, where *n* and *m* are the image height and width in pixels, and 3 is the number of color channels. In a convolutional neural network, image features are extracted from a volume by a series of convolutional layers, which learn collections of filters. These filters are applied pixel-wise in strided convolutions (in a sliding window fashion) over the input volume, where the dot product between the filter weights and each spatial location (assuming a stride size of one pixel) in the input volume creates an activation map. Similarly, the output volume of the convolutional layer is an $p \times q \times k$ volume where *p* and *q* are some spatial extents, and *k* represents the number of filters in the layer (and therefore the number of filter activation maps). As with regular neural network layers, a non-linear function is applied to the activations.

In order to construct a hierarchical representation of the data, many convolutional layers are alternated with pooling layers, which downsample the spatial size of the input volume. The output of the final convolutional layer (or final pooling layer) represents a learned representation of the original input data. This learned representation is used by fully-connected neural network layers to perform classification or regression, and all of the network’s parameters are learned simultaneously during training. A more detailed overview of CNNs for plant scientists is provided in [6], and readers may refer to the deep learning literature for more technical descriptions [5].

For some applications, the construction of large data sets of labeled images can be facilitated by crowd-sourcing images freely available on the Internet [4]. Unfortunately, this approach is not possible for plant phenotyping datasets, due to their specificity. The creation of these datasets requires sampling a wide range of accessions, and many individual plants need to be cultivated from germination to maturity. Along with the agricultural work involved, each plant must be imaged individually (or segmented from a tray image containing multiple plants), and each image needs to be annotated with ground truth data, measured manually and/or specified by an expert. Although high-throughput imaging systems do exist to expedite the process of collecting large sets of plant images, the end-to-end phenotyping process remains prohibitively time consuming and expensive, limiting the size of the available datasets. Existing plant image datasets are available for a wide range of applications, including both roots and shoots [10]. These public collections are a valuable source of data for many applications, and often do include annotations for ground truth. However, we find it compelling to offer a source of new, additional data alongside these public collections which is free of the aforementioned limitations.

Even for large training datasets, the network can still fail to properly recognize phenotypes if the distribution of testing data differs significantly from that of the training data. In the case of leaf counting, the distribution of leaf numbers in the training data must be similar to that of the testing data: if the rosettes used for training have significantly fewer leaves than the rosettes used for testing, the learned model will likely be *misspecified* and mis-predict the number of leaves. In technical terms, the learning process infers a conditional model *P*(*y*|*x*): the conditional distribution of the outputs given the inputs. Differences between training and testing data can result in two related problems known as *covariate shift*, where *P*(*x*) changes between training and testing, and *dataset shift*, a different joint distribution *P*(*x*, *y*) of the outputs and inputs in the test data, compared to that in the training data. This problem is common in machine learning and can be difficult to mitigate [11]. Available techniques often focus on statistically modeling the difference between the training and testing distributions. However, finding such a mapping is not only practically infeasible for complex vision-based tasks, but also assumes the availability of samples drawn from the test distribution. These issues are unique to supervised learning, as hand-engineered pipelines containing *a priori* information typically do not have to model the conditional distribution explicitly. The problem of dataset shift is almost inevitable when using supervised learning for plant phenotyping tasks, due to the limitations of generating new plant phenotyping datasets. It is not possible to specify the domain of phenotypes to be represented in the data, and so this limitation will tend to expose problems of dataset shift when using models of phenotypes learned from this data. We investigate the use of computational plant models to mitigate this problem.

### Computational plant models

Computational modeling has become an inherent part of studies of plant physiology, development, architecture, and interactions with the environment. Diverse concepts and techniques exists, applicable to construct models at spatio-temporal scales ranging from individual cells to tissues, plant organs, whole plants, and ecosystems [12–14]. The formalism of L-systems [15], augmented with a geometric interpretation [16, 17] provides the basis for a class of specialized programming languages [17–19] and software (e.g. [20–22]) widely used to model plants at different levels of abstraction and for a variety of purposes. In the domain of phenotyping, Benoit et al. [23] employed an L-system-based root model [24] to generate testing data for validating image-based root system descriptions. To create or augment training data sets for image-based leaf counting tasks considered in this paper, we constructed a descriptive model that reproduces early developmental stages of the plant shoot on the basis of direct observations and measurements (without accounting for the underlying physiological processes). Applications of L-systems to construct such models are presented, for example, in [17]; the subsequent enhancements include gradual modifications of the organ shapes as a function of their age [25, 26] and position in the plant [27], as well as the use of detailed measurements of shape [28]. The model of rosettes used in this paper is the first application of L-systems to model plant shoots for phenotyping purposes.

### Related work

The use of synthetic or simulation data has been explored in several visual learning contexts, including pose estimation [29] as well as viewpoint estimation [30]. In the plant phenotyping literature, models have been used as testing data to validate image-based root system descriptions [23], as well as to train machine learning models for root description tasks [31]. However, when using synthetic images, the model was both trained and tested on synthetic data, leaving it unclear whether the use of synthetic roots could offer advantages to the analysis of real root systems, or how a similar technique would perform on shoots.

The specialized root system models used by Benoit et al. [23] and Lobet et al. [31] are not applicable to tasks involving the aerial parts of a plant—the models have not been generalized to produce structures other than roots. Nonetheless, for image-based tasks Benoit et al. [23] were the first to employ a model [24] based on the L-system formalism. Because of its effectiveness in modelling the structure and development of plants, we chose the same formalism for creating our Arabidopsis rosette model

## Methods

In the present work, we seek to demonstrate that realistic models of synthetic plants are a sufficient replacement for real data for image-based plant phenotyping tasks. We show that a model of the *Arabidopsis thaliana* rosette can be used either in conjunction with real data, or alone as a replacement for a real dataset, to train a deep convolutional neural network to accurately count the number of leaves in a rosette image. We also discuss how the concept of model-based data augmentation may extend to other plants and phenotyping tasks.

### Image sources and processing

For the images of real plants used in the leaf counting task, we use a publicly available plant phenotyping dataset from the International Plant Phenotyping Network (IPPN),^1^ referred to by its authors as the PRL dataset [32]. The PRL dataset is a multi-purpose phenotyping dataset that includes ground truth labels for several different phenotyping tasks, including leaf counting and segmentation, age estimation (hours after germination), and mutant classification. Two annotated image subsets are available within PRL for the leaf counting task using Arabidopsis rosettes considered in this paper. These subsets, referred to as Ara2012 and Ara2013-Canon, vary in the several ways, including the accessions of the subjects, lighting, level of zoom, image sizes, leaf size and shape, and the distributions of the number of leaves (Table 1). The full datasets, as well as several alternative versions, are downloadable at https://figshare.com/articles/SATLC-28-09-17_zip/5450080.

**Table 1:** Real and synthetic training datasets

Dataset	Number of images	Range of leaf counts	Accessions	Image size	Scale$^{\mathrm{a}}$	Background
Ara2012	120	12–20	Col-0	Varied	1:1	Soil/tray
Ara2013-Canon	165	5–13	Col-0/mutants	Varied	1:1	Soil
S1	1000	12–20	N/A	$256 \times 256$	1:1–1:2	Soil
S2	1000	5–13	N/A	$256 \times 256$	1:1–1:2	Soil
S12	1000	5–20	N/A	$256 \times 256$	1:1–1:2	Varied

$^{\mathrm{a}}$ Scale denotes the ratio of the plant diameter to the image size

When training on synthetic images and testing on real images (as in Table 3 rows 3, 4, and Table 4 rows 1, 3), we set the background pixels to black using the segmentation masks provided with the PRL dataset. This was done to prevent the network from reacting to objects in the background of the image, which were not accounted for in the plant model. Although training on images of real plants with a variety of non-uniform backgrounds results in a model which is conditioned to be invariant to such backgrounds, these backgrounds are more difficult to control for when using synthetic plants as the training data. Although we use the foreground-background segmentations provided by the authors of the dataset, automatic segmentation methods targeting plants [33–35] or general-purpose [36] could also be considered.

### CNN architectures

In the augmentation experiment, we replicated the architecture used in conjunction with the Ara2013-Canon dataset in the reference experiment [6], in order to compare our results with those published previously. This architecture uses three convolutional layers, each with a $5 \times 5$ spatial resolution and a stride size of one pixel, and each followed by a $3 \times 3$ pooling layer with a stride size of two pixels. In the remaining experiments (generalization and interoperability), we employed a larger CNN architecture, used in conjunction with the Ara2012 dataset in [6]. This architecture uses four convolutional layers, each followed by a pooling layer, and a single fully connected layer with 1024 units, followed by the output layer. The *tanh* activation function was used in all cases, and $\lambda = 10^{-4}$ was used for the L2 weight decay when training on synthetic data to limit overfitting. In all experiments, the static learning rate was $10^{-3}$. The training dataset was augmented with standard image-based techniques. Image variation was increased using vertical and/or horizontal flips, and cropping by 10% to a window randomly positioned within the input image. The brightness and contrast were also randomly modified. As in previous work, we split the data randomly into training (80%) and testing (20%) for each experiment.

### An L-system model of the Arabidopsis rosette

To augment the PRL dataset of Arabidopsis rosette images, we developed a model of Arabidopsis in the vegetative stage based on an existing model [28]. The model was implemented using the L-system-based plant simulator lpfg included in the Virtual Laboratory plant modeling environment [20, 37]. The full model code is available in the dataset file which has been provided for download. The rosette was constructed as a monopodial structure with leaves arranged on a short stem in a phyllotactic pattern. The length of a leaf, $l_n(t)$, at node number *n* and age *t* was computed as $l_n(t) = f_{lmax}(n) \cdot f_{l}(t)$, where $f_{lmax}(n)$ is the final length given the node number, and $f_{l}(t)$ controls the leaf length over time. Leaf blades were modeled as flat surfaces, fitted to an arbitrarily chosen image of an Arabidopsis leaf from the Ara2012 dataset. The width of the leaf blade was scaled proportionally to its length, $w_n(t,x) = l_n(t) \cdot f_{lw}(x)$, where $f_{lw}(x)$ is the leaf contour function and *x* is the distance from the leaf base along the midrib. Petiole length was set to be proportional to leaf length, and petiole width was assumed to be constant. The leaf inclination angle was specified as a function of node number $f_{ang}(n)$.

**Fig. 1:**
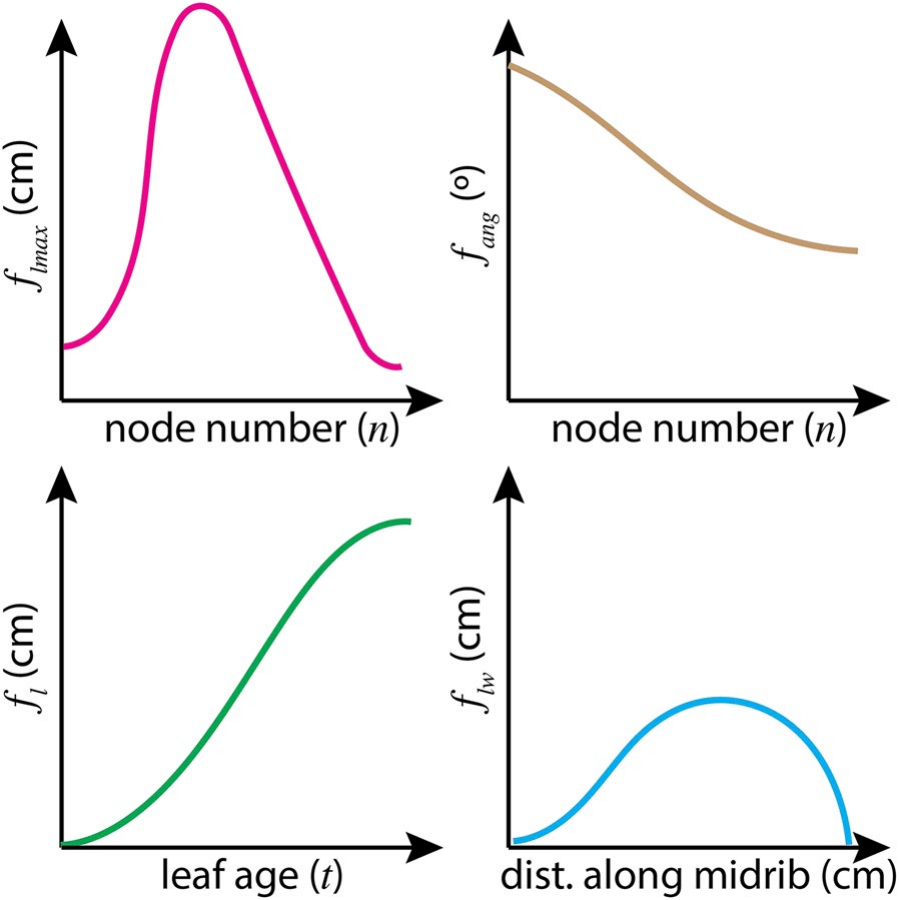
Leaf growth and shape functions used in the L-system model

All functions were defined using the Virtual Laboratory graphical function editor funcedit (Fig. 1). The shapes of the functions were drawn (by manual placement of control points) such that the final leaf length, leaf length over time, inclination angle, and leaf shape agreed with the published measurements [28].

We modeled the diversity of Arabidopsis rosettes by modifying the final leaf length (and, proportionally, the leaf width) using normally distributed random variables. Specifically, for each leaf along the stem, we multiplied $f_{lmax}(n)$ by a variable $X_n$ taken from normal distribution with mean $\mu =1$ and standard deviation $\sigma =10^{-2}$. Likewise, the divergence (phyllotactic) angle between consecutive leaves *n* and $n+1$ was calculated as a normally distributed random variable $\theta _n$ with mean $\mu =137.5$ and standard deviation $\sigma =2.5$. Finally, the time of development of the rosette was varied using a uniform random variable for each simulation run, such that the final number of leaves was in the range from 5 to 20.

**Fig. 2:**
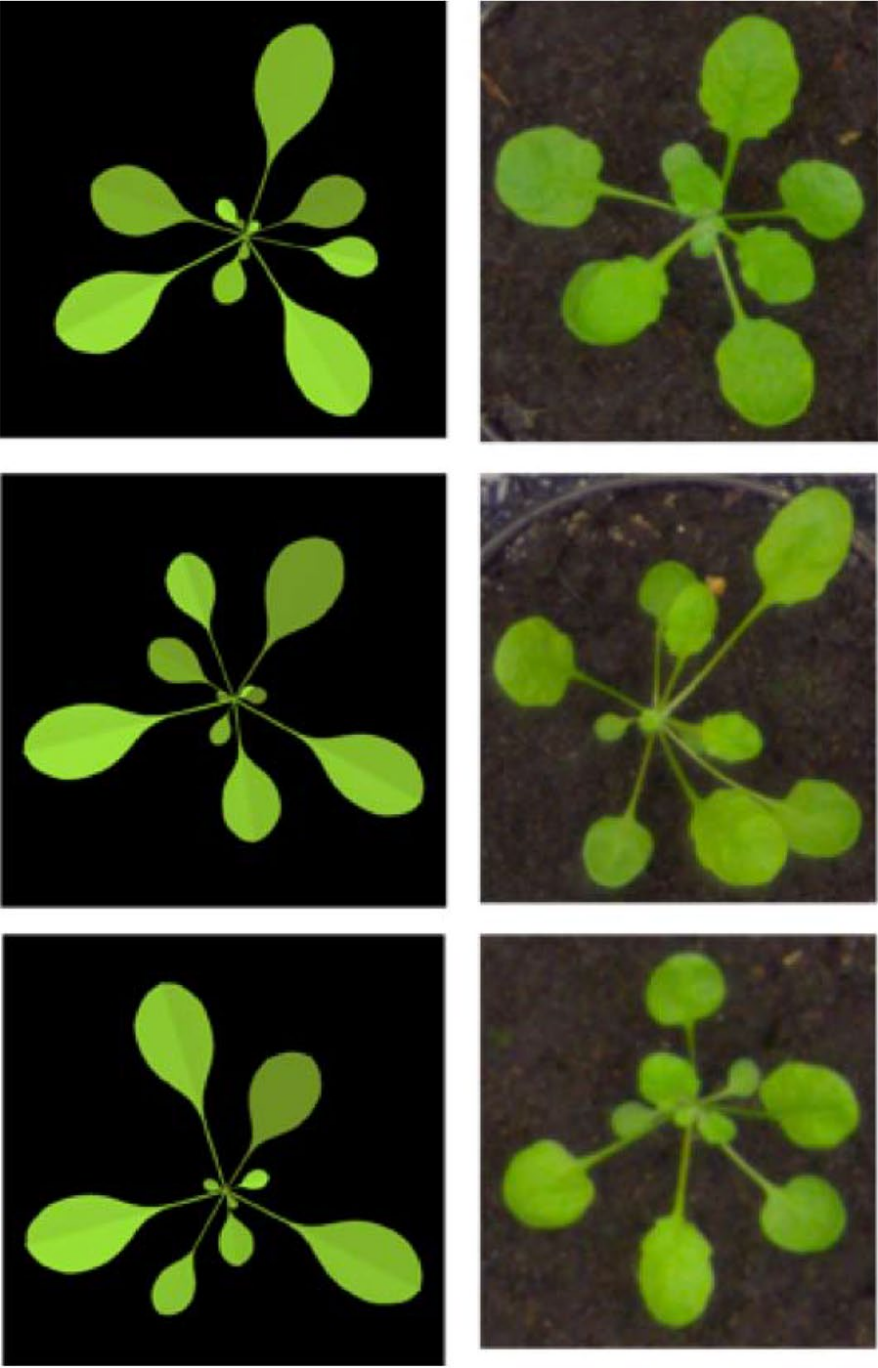
Synthetic rosettes (left) generated by the L-system and real rosettes (right) from the public dataset [32]

Our model was implemented using parametric L-systems, in which each component of a plant (apex, leaf, and internode) has a corresponding module with associated parameters [17]. For example, in the module A(*n*) representing the apex, the parameter *n* is the node number. We simulated the development of the plant by a set of rewriting rules, which specify the fate of each module (component) over an increment of time. An apex, for instance, produces a new internode and new leaf at regular time intervals. To account for diversity of rosettes, we generated 1000 images with a random variation. Details of our implementation are given in the Additional file 1. Figure 2 shows three example renderings alongside three real images for visual comparison.

## Results

To validate the use of models with deep learning, we conducted three leaf counting experiments using images of both real and synthetic Arabidopsis rosettes. The mean absolute count difference, and the standard deviation of absolute count difference, were measured in each experiment. The experiments were conducted as follows:

### Augmentation

This experiment tested the usefulness of synthetic plants in augmenting the Ara2013-Canon dataset of real plants for the leaf counting task. For this purpose, we generated a set of one thousand synthetic rosettes (S2) and added them to the training set. The model’s background was set to a brown color approximating the soil in the real dataset. Using synthetic rosettes to augment the training set, we observed a reduction of approximately 27% in the mean absolute count error (Table 2).

**Table 2:** Augmentation results, Ara2013-Canon dataset

	AbsCountDiff	CountDiff	MSE	$R^2$	Agreement (%)
Ubbens and Stavness [6]	0.61 (0.52)	–	–	–	–
Synthetically augmented (S2)	0.48 (0.58)	0.15 (0.82)	0.73	0.92	80

### Generalization

In this experiment we investigated whether the ability of the model to generate an arbitrary range of phenotypes may be used to mitigate the problem of dataset shift. To this end, we trained a leaf counting network on purely synthetic data and tested it on two real datasets, each with a different distribution of leaf numbers. These datasets exhibit both covariate shift in the different distributions of leaf counts, as well as dataset shift in the intersection between the two as described in the background on deep learning. For brevity, we will address both problems as dataset shift in our discussion. The synthetic training data consisted of one thousand synthetic rosettes with a uniform distribution of leaf numbers between five and twenty (S12). The model was then tested on the Ara2012 dataset (with a range of between 12 and 20 leaves) and the Ara2013-Canon dataset (between 5 and 13 leaves). A synthetic training set which is easy for the network to fit will result in poor generalization due to overfitting; in order to introduce more variance to the synthetic data with the goal of reducing overfitting, the model’s background was set to either a soil color or a random color in RGB space ($p=0.5$). Although the images the network was tested on were segmented onto a black background, the addition of different background colors in the model varied the contrast between the leaves and background in the individual color channels, which showed to be beneficial for generalization when using synthetic images.

**Fig. 3:**
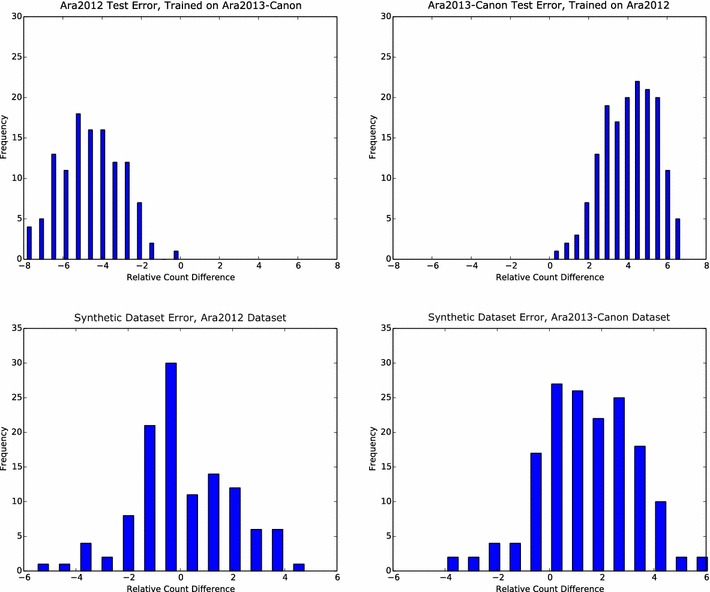
Distributions of relative count difference in the generalization experiment. Training on one dataset and testing on another exhibits severe dataset shift (top), while training on synthetic data significantly reduces this error by encompassing a comprehensive range of leaf counts (bottom)

When training on dataset Ara2012 and testing on Ara2013-Canon, or vice versa, we observed significantly degraded performance due to dataset shift. However, when training on a purely synthetic rosettes, dataset shift is mitigated with mean count error more closely centered around zero (Table 3). The distributions of relative count errors for both real datasets when trained on real and synthetic data are shown in Fig. 3. Although the mean absolute count errors are similar in each case, the coefficient of determination shows that the predictions made on Ara2012 are much more strongly correlated with the ground truth measurements ($R^2=0.42$) than those on Ara2013-Canon ($R^2=-0.33$).

**Table 3:** Performance when training and testing on different datasets.

Training data	Testing data	AbsCountDiff	CountDiff	MSE	$R^2$	Agreement (%)
Ara2013-Canon	Ara2012	5.45 (2.04)	$-$ 5.45 (2.04)	33.9	$-$ 4.79	0
Ara2012	Ara2013-Canon	5.39 (1.99)	5.39 (1.99)	33.13	$-$ 6.15	0
S12	Ara2012	1.38 (1.03)	$-$ 0.25 (1.7)	2.97	0.42	22
S12	Ara2013-Canon	1.82 (1.38)	0.46 (2.24)	5.25	$-$ 0.33	20

Training on a single dataset of synthetic rosettes performs significantly better than training on a dataset of real rosettes with a different distribution of phenotypes

### Interoperability

**Fig. 4:**
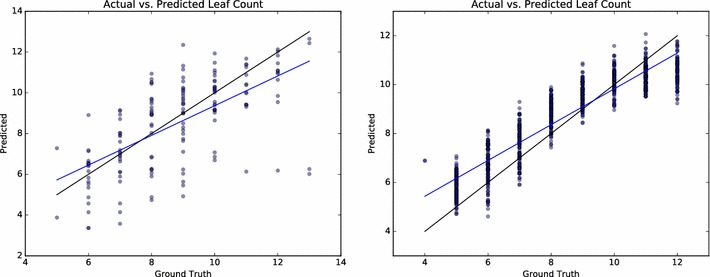
Scatter plots of actual and predicted leaf counts in the interoperability experiments. Training on synthetic and testing on real (left), and training on real and testing on synthetic (right)

This experiment tested the interoperability between real and synthetic plants by training a network on real plants (Ara2013-Canon) and testing it on synthetic plants (S2) containing the same range of leaf numbers, or vice versa: training on the set S2 and testing on Ara2013-Canon. A small error value in this experiment signifies that the model is a suitable stand-in for real plants for the leaf counting task. Statistics are provided for both cases (Table 4), as well as scatter plots illustrating the correlation between ground truth and predicted value (Fig. 4). Although the $R^2$ statistics are substantially lower when using synthetic data, this is partially due to a small number of outliers which are highly penalized due to the squared error term in the $R^2$ calculation. The scatter plots (Fig. 4) show these outliers as well as a line of best fit, which shows better correlation with ground truth than the $R^2$ statistics would suggest.

**Table 4:** Interoperability between real and synthetic rosettes

Training data	Testing data	AbsCountDiff	CountDiff	MSE	$R^2$	Agreement (%)
S2	Ara2013-Canon	1.29 (1.01)	$-$ 0.02 (1.64)	2.7	0.26	24
Ara2013-Canon	S2	0.81 (0.54)	0.28 (0.93)	0.95	0.82	34
S1	Ara2012	1.70 (1.21)	0.67 (1.98)	4.39	0.27	25

## Discussion

Deep learning models, including the deep CNNs used in the experiments presented here, have a large capacity for fitting the training data. This is essential to their learning ability, but also makes them susceptible to overfitting in the case of small datasets, or large datasets with an insufficient level of variation. Therefore, it is important to consider how to introduce as much variation as possible into the model and the scene. For example, we found that generalization improved when plants were randomly scaled, with the ratio of the plant diameter to the size of the entire image varying between 1:1 and 1:2. This helped prevent the network from using the number of green pixels as a proxy for the number of leaves, which could be a viable strategy if the model lacked enough variance in leaf size. Other considerations include varying the contrast between background and foreground pixels. Such variations in the model, the scene, as well as secondary image-based augmentations such as modifications of the brightness and contrast all contribute to preventing overfitting.

**Fig. 5:**
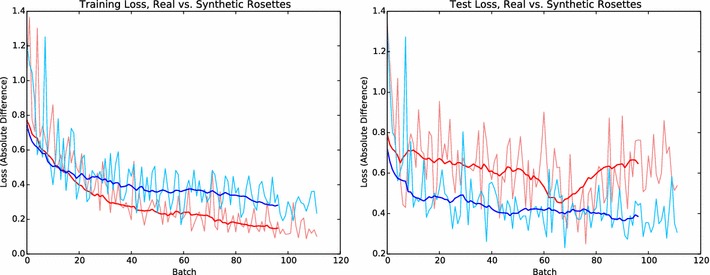
Comparison of training and testing loss on real (red) and synthetic (blue) rosettes. Real plants show significantly higher generalization error, while the synthetic dataset is relatively easy to fit

**Fig. 6:**
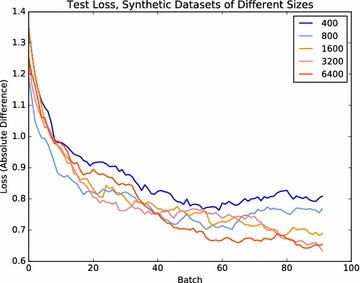
Test performance on purely synthetic data when using increasing sizes for the training set. Like with datasets of natural images, we see that generalization performance improves with larger training sets

Comparing the counting errors during training and testing, we observed that their difference (the generalization error) is larger for real data than for synthetic data (Fig. 5). This means that, despite attempts to capture specimen-to-specimen variation using a stochastic model, our synthetic plants are significantly easier to fit and therefore do not fully capture the diversity of real rosettes. The network’s performance in the task of counting real leaves could thus be improved by adding more variation to the set of synthetic plants used for training. However, even with the limited variation, networks trained on the synthetic rosettes do seem to benefit from larger training sets (Fig. 6), which is a characteristic typically seen in natural datasets as well.

Another consequence of overfitting is the network’s tendency to discriminate between different types of data. In tests with both real and synthetic data, if these datasets had different leaf distributions, the network would learn to map each type of data to an individual output distribution, with a detrimental effect on generalization performance. This means that the use of synthetic data in conjunction with real data is only advisable if the distributions of phenotypes of the real and synthetic data overlap. Although this could be seen as a disadvantage, we have also shown that the use of synthetic data alone is sufficient and avoids this effect.

We observed that models which are not sufficiently realistic resulted in degraded performance compared to more accurate models. For example, an initial rosette model in which all leaves were assumed to be of the same size showed significantly lower interoperability with the images of real rosettes. Taking into account not only the differences in leaf size, but also in shape as a function of their position [28], as well as capturing differences in leaf colour and texture, may further contribute to the realism and diversity of synthetic images used for training purposes. Future work includes the inclusion of a more detailed model of leaf shape which includes serrations and sinuses. These considerations were not included in the present model due to limited variance in leaf shape in the available images of real rosettes. Ultimately, the most accurate images of plants under different conditions may be provided by mechanistic models relating plant appearance to the underlying physiological processes.

Future directions for research could further explore the relationship between models trained on real data and those trained on synthetic data, including techniques such as transfer learning. Using a feature extractor learned on synthetic data and re-training a regressor with these features may shed light on differences in learned representations between the two types of data.

In summary, the results presented in this paper show promise for the use of models in image-based plant phenotyping tasks. The existing body of work on L-system modeling of plants is extensive, with models available for many different species. These existing models are well positioned to take the results demonstrated here on Arabidopsis forward towards other applications. One potentially important application area is the modeling of entire plots of crops. A simulated plot of plants could potentially make it possible to train algorithms for detecting biologically meaningful traits such as flowering time or response to stress with a reduced number of real (annotated) crop images. Other directions for future work could include augmentation using synthetic data for other supervised learning problems, such as leaf segmentation. Other applications, such as disease detection, would be possible if future plant models were able to model such phenomena.

## Conclusion

We applied a computer-generated model of the Arabidopsis rosette to improving leaf counting performance with convolutional neural networks. Using synthetic rosettes alongside real training data, we reduced mean absolute count error with respect to results obtained previously using only images of real plants [6]. We also demonstrated that—due to the model’s ability to generate an arbitrary distribution of phenotypes—a network trained on synthetic rosettes can generalize to two separate datasets of real rosette images, each with a different distribution of leaf counts. Finally, the interoperability experiments have shown, in particular, that a CNN trained only on synthetic rosettes can be successfully applied to count leaves in real rosettes. 3D plant models are thus useful in training neural networks for image-based plant phenotyping purposes.

## Additional file

**Additional file 1.** The complete code of the *Arabidopsis thaliana* rosette model L-system.

## Footnotes

1
https://www.plant-phenotyping.org/datasets-home.
