# Supplementary material for: The use of plant models in deep learning: an application to leaf counting in rosette plants
Source: Plant Methods. 2018 Jan 18;14:6. doi: 10.1186/s13007-018-0273-z (PMC5773030; doi:10.1186/s13007-018-0273-z)
Supplement: Supplementary file 1 — Additional file 1. The complete code of the Arabidopsis thaliana rosette model L-system. [file 13007_2018_273_MOESM1_ESM.pdf]

RESEARCH

Supporting Information: The use of plant models in deep learning: an application to leaf counting in rosette plants

Jordan Ubbens[1], Mikolaj Cieslak[2], Przemyslaw Prusinkiewicz[2] and Ian Stavness[1*]

[*]Correspondence:
ian.stavness@usask.ca
[1]University of Saskatchewan, 105 Administration Place, S7N 5C5 Saskatoon, Canada
Full list of author information is available at the end of the article

Model implementation

Our Arabidopsis rosette model was specified in the L+C programming language [1, 2], which combines L-systems [3] and the C++ programming language. The model was executed using the *lpfg* simulator [1] included in the Virtual Laboratory software environment [4]. The source code for our model is given in Listing 1, and an *lpfg*-generated image of the model is shown in Figure S1.

Listing 1 L+C implementation of the Arabidopsis rosette model.

```
#include <lpfgall.h>

// Declaration of constants
const float $n_{max} = 20.$; // max. number of leaves
const float $\Delta a = 0.1$; // leaf age increment
const float $t_{min} = 300.$; // min. growing time
const float $t_{max} = 820.$; // max. growing time
const float $d_{age} = 4.5$; // min. drawing age

// Module definitions
module A(float);        // an apex, with node number
module L(float,float); // a leaf, with node number and age

// Setup time of development and leaf counting
int $s$; // counts simulation steps
int $s_{max}$; // last simulation step
int $N$; // total number of leaves

Start: {
  $s = 0$;
  $s_{max} =$ int($t_{max}$ * ran(1)) + $t_{min}$;
}
StartEach: {
  $N = 0$;
}
EndEach: {
  $s = s + 1$;
  if ($s$ == $s_{max}$) {
    Stop();
    Printf ("Leaf count: %d\n", final_num_leaves);
  }
}
```

```

derivation length: 10000;

axiom: Right(nran(0,5.0)) RollL(nran(0,180)) A(0);

decomposition:

A(n) : {
  if (n < n_max) {
    produce SetWidth(10^-2) F(10^-3) // internode
            SB L(n,0) EB // leaf
            RollL(nran(137.5,2.5)) // phyllotaxis
            A(n+1); // recreate apex
  }
}

production:

L(n,a) : {
  a = a + Δa;
  // count leaf if above a certain age
  if (a > d_age)
    N = N + 1;
  produce L(n,a);
}

interpretation:
// declare extra module for drawing a leaf
module C(float,float,bool);

L(n,a): {
  // do not visualize very small leaves
  if (a ≤ d_age)
    produce;

  // calculate the leaf's length
  float l_n = nran(1.,0.01) * f_lmax(n/nran(1.,0.05)) * f_l(a);

  // produce the leaf's geometry
  produce Down(45 * f_ang(n)) // set inclination angle
          CurrentContour(1) // specify leaf cross-section
          SetWidth(l_n * f_w(0)) // set width at leaf base
          StartGC C(0,0.65 * l_n,0) EndGC // draw petiole
          StartGC C(0,l_n,1) EndGC; // draw leaf
}

C(x,l,q) : {
  const float Δx = 0.01; // step size along midrib
  if (x ≤ 1) {
    x = x + Δx;
    float w = l;
    if (q)
      w = w * f_w(x);
    else
      w = w * f_w(0)/0.65;
    nproduce F(l * Δx) SetWidth(w) Down(15 * Δx) C(x,l,q);
  }
  produce;
}
```

After declaration of constants (lines 4–8) and module definitions (lines 11–12), global variables are declared for the number of steps in the simulation and the total number of leaves in the rosette (lines 15–17). At the start of the simulation, the number of steps is initialized to zero and the maximum number of steps is computed as a discrete random uniform variable in the range $[t_{min}, t_{max}]$ (lines 20–21). After each derivation step, the number of steps is incremented until the maximum is reached, which ends the simulation and prints the total leaf count (lines 27–30).

A single apex, A, is initiated in the axiom (line 36) after some randomization of the rosette's orientation. The first production generates a new internode and leaf up to a maximum number n_{max} by decomposing the module A (lines 40–45). Each internode is modeled as a cylinder (module F) with constant radius 10^{-2} and length 10^{-3}. Consecutive leaves are rotated around the main stem following a spiral phyllotaxis by an angle generated from a normal random distribution with mean 137.5° and standard deviation 2.5. The second production (lines 51–56) models a leaf's aging and increments the global leaf counting variable, N, if the leaf is above a certain age.

To generate a visualization of the rosette, we apply interpretation rules to the string generated by the L-system (starting from line 59). The first interpretation rule is applied to all leaf modules $\mathrm{L}(n, a)$. The rule first ignores a leaf (produces nothing) if its age is below a threshold. Then, given the leaf's node number and age, the leaf's length (line 69) is calculated as described in the main text. The rule ends by starting the drawing of a generalized cylinder, module $\mathrm{C}(x, l, q)$ representing the leaf, which is separated into the petiole and lamina sections (lines 72–76).

The interpretation rule for module $\mathrm{C}(x, l, q)$ (line 79) draws consecutive cross sections of the leaf along the midrib. This is effected by applying the rule recursively until the drawing is forced to stop (line 81) — when the variable x is greater than one. The width of the generalized cylinder is determined by its length l and its state variable q. For the lamina ($q = 1$), the width is modified by a graphically defined function, $f_w(x)$, as the cylinder is being drawn, but, for the petiole ($q = 0$), the width is constant (using the value at $f_w(x = 0)$). Production of the $\mathrm{F}(l * \Delta x)$ and $\mathrm{SetWidth}(w)$ modules (line 88) draws a section of the generalized cylinder with the given length and width. The cylinder is also rotated downwards by $(15 * \Delta x)°$ after each section is drawn.

Author details
[1]University of Saskatchewan, 105 Administration Place, S7N 5C5 Saskatoon, Canada. [2]University of Calgary, 2500 University Dr NW, T2N 1N4 Calgary, Canada.

References

1. Karwowski, R., Prusinkiewicz, P.: Design and implementation of the L+C modeling language. Electronic Notes in Theoretical Computer Science **86**(2), 1–19 (2003). doi:10.1016/S1571-0661(04)80680-7
2. Prusinkiewicz, P., Karwowski, R., Lane, B.: The L+C plant-modelling language. In: Vos, J., Marcelis, L., De Visser, P., Struik, P., Evers, J. (eds.) Functional–structural Plant Modelling in Crop Production, pp. 27–42. Springer, Dordrecht (2007)
3. Prusinkiewicz, P., Lindenmayer, A.: The Algorithmic Beauty of Plants. Springer-Verlag, New York (1990). With Hanan J, Fracchia FD, Fowler D, de Boer MJM, and Mercer L.
4. Federl, P., Prusinkiewicz, P.: Virtual Laboratory: an interactive software environment for computer graphics. In: Proceedings of Computer Graphics International, pp. 93–100 (1999)

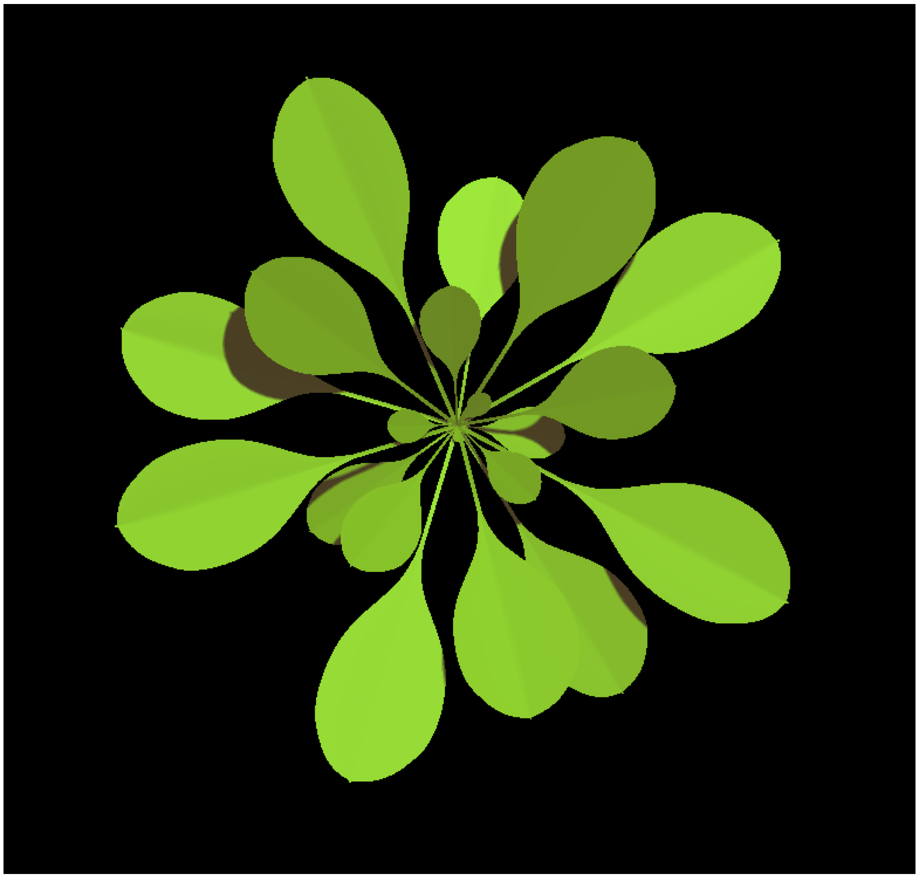

Figure S1 An example rendering of the Arabidopsis rosette model with 20 leaves.
